# Involvement of KLF11 in Hepatic Glucose Metabolism in Mice via Suppressing of PEPCK-C Expression

**DOI:** 10.1371/journal.pone.0089552

**Published:** 2014-02-26

**Authors:** Huabing Zhang, Qi Chen, Tao Jiao, Anfang Cui, Xiujing Sun, Weijun Fang, Liwei Xie, Yang Liu, Fude Fang, Yongsheng Chang

**Affiliations:** 1 Department of Biochemistry & Molecular Biology, School of Basic Medicine, Anhui Medical University, Hefei, China; 2 National Laboratory of Medical Molecular Biology, Institute of Basic Medical Science, Chinese Academy of Medical Science and Peking Union Medical College, Beijing, China; 3 Department of Biochemistry, Jining Medical University, Jining, China; 4 Beijing Friendship Hospital, Capital Medical University, Beijing Key Laboratory for Precancerous Lesion of Digestive Diseases, Beijing Digestive Disease Center, Beijing, China; 5 Department of Chemistry, School of Basic Medicine, Anhui Medical University, Hefei, China; 6 Integrative and Molecular Physiology, University of Michigan, Ann Arbor, Michigan, United States of America; 7 Department of Endocrinology, The Hospital Affiliated to Liaoning University of Traditional Chinese Medicine, Shenyang, China; Institut d’Investigacions Biomèdiques August Pi i Sunyer, Spain

## Abstract

**Background:**

Abnormal hepatic gluconeogenesis is related to hyperglycemia in mammals with insulin resistance. Despite the strong evidences linking Krüppel-like factor 11 (KLF11) gene mutations to development of Type 2 diabetes, the precise physiological functions of KLF11 *in vivo* remain largely unknown.

**Results:**

In current investigation, we showed that KLF11 is involved in modulating hepatic glucose metabolism in mice. Overexpression of KLF11 in primary mouse hepatocytes could inhibit the expression of gluconeogenic genes, including phosphoenolpyruvate carboxykinase (cytosolic isoform, PEPCK-C) and peroxisome proliferator–activated receptor γ coactivator-1α (PGC-1α), subsequently decreasing the cellular glucose output. Diabetic mice with overexpression of KLF11 gene in livers significantly ameliorated hyperglycemia and glucose intolerance; in contrast, the knockdown of KLF11 expression in db/m and C57BL/6J mice livers impaired glucose tolerance.

**Conclusions:**

Our data strongly indicated the involvement of KLF11 in hepatic glucose homeostasis via modulating the expression of PEPCK-C.

## Introduction

Normal blood glucose levels are tightly maintained within a narrow range by a sophisticated regulatory system to provide a constant fuel supply for the body. The liver plays a critical role in the maintenance of systemic glucose homeostasis. Hepatic gluconeogenesis, the net production of glucose from substrate molecules, is critical for the adaptation to fasting conditions [1], [2]. However, abnormal activation of hepatic gluconeogenesis contributes to hyperglycemia [3]. In the absorptive state, ingested glucose is taken up by hepatocytes and converted to glycogen and lipids. In the postabsorptive state, hepatocytes produce glucose, which is secreted into the circulation. Insulin and counter-regulatory hormones (e.g. glucagon and glucocorticoids) regulate hepatic glucose production mainly by regulating the hepatic gluconeogenic program [4]. Regulation of gluconeogenesis in the liver is thought to be achieved through control of the expression of genes encoding gluconeogenic enzymes such as phosphoenolpyruvate carboxykinase (cytosolic isoform, PEPCK-C) and glucose-6-phosphatase (G6Pase) [5]. Insulin decreases hepatic glucose production by suppressing the expression of key gluconeogenic genes; conversely, counter-regulatory hormones increase hepatic glucose production by stimulating the transcription of these genes [1]. Multiple transcription factors, including cAMP-responsive element–binding protein (CREB), and forkhead factor O1 (FoxO1), as well as transcriptional coactivators such as CREB binding protein (CBP), and peroxisome proliferator–activated receptor γ (PPARγ) coactivator-1α (PGC-1α) have been identified to regulate the expression of the gluconeogenic genes in the liver [6]–[9].

The Krüppel-like family of transcription factors is a subclass of Cys2/His2 zinc-finger DNA-binding proteins [10]. Krüppel-like factors (KLFs) are a critical regulators of the growth and development in a wide variety of tissues [11], [12]. The members of this protein family contain three C2H2 zinc fingers near their C-terminus, which recognize CACCC and related GC-rich elements in promoters and enhancers, and their N-terminal domains are highly variable and show different molecular functions [12]. KLF11 is expressed ubiquitously, with high expression levels in the pancreas and plays a key role in the regulation of pancreatic beta cell physiology, and its variants may contribute to the development of diabetes [13], [14]. Additionally, *Klf11^−/−^* mice recapitulate the disruption in insulin production and high blood glucose levels observed in diabetic patients [15]. These observations raise the possibility that KLF11 may be involved in the regulation of glucose and lipid metabolism.

Previously, we have demonstrated that hepatic *KLF11* gene expression was regulated by nutritional status and dysregulated in diabetic and diet-induced obesity (DIO) mice. Moreover, overexpression of KLF11 in the livers of db/db and DIO mice activated the peroxisome-proliferator-activated receptor α (PPARα) signaling pathway and markedly improved the fatty liver phenotype [16], suggesting that KLF11 is an important regulator of hepatic lipid metabolism. We also found that overexpression of KLF11 in livers of db/db diabetic mice decreased fasting blood glucose levels [16], however, the underlying molecular mechanisms of its action have not been explored.

In this study, we have investigated the roles of KLF11 in the regulation of the hepatic gluconeogenic programs. We showed that adenovirus-mediated overexpression of KLF11 in livers of db/db diabetic mice alleviated hyperglycemia and glucose intolerance. Hepatic silencing of KLF11 impaired glucose homeostasis in db/m and wild-type C57BL/6J mice. In addition, we found that KLF11 inhibited cellular glucose production in primary hepatocytes by directly suppressing transcription of *PEPCK-C* gene. These data supported that the KLF11 gene is an important physiological regulator of hepatic gluconeogenesis.

## Materials and Methods

### Animals and Experimental Design

Male db/db, db/m and C57BL/6J mice at 8–9 weeks of age were purchased from the Model Animal Research Center of Nanjing University (Nanjing, China) and housed and maintained on a 12 hr light-dark cycle with a regular unrestricted diet. Wild-type C57BL/6 mice were fed either normal chow (9% fat; Lab Diet) or a high-fat (HF) diet (45% fat; Research Diets, NB, USA) *ad libitum* with free access to water. All animal experiments were conducted under protocols approved by the Animal Research Committee of the Institute of Laboratory Animals, Chinese Academy of Medical Sciences and Peking Union Medical College. Mice were injected intravenously through the tail vein with Ad-KLF11, Ad-shKLF11, Ad-GFP or Ad-shControl (Control) (0.5–1.0×10^9^ active viral particles in 200 µl of PBS). Then, 5–7 days after infection, mice were fasted for 6 hrs (from 8∶00 a.m. to 2∶00 p.m.) and their livers were collected for further analysis. For glucose tolerance tests (GTTs) or pyruvate-tolerance tests (PTTs), mice were fasted overnight (16 hrs), and D-glucose or pyruvate was injected intraperitoneally at a dose of 2 g/kg. For insulin tolerance tests (ITTs), mice were fasted for 6 hrs (from 10∶00 a.m. to 4∶00 p.m.) and human insulin was injected intraperitoneally at a dose of 0.5 U/kg for db/m and C57BL/6J mice and 0.75 U/kg for db/db mice. Blood glucose levels were measured from the tail vein at indicated times using a glucometer (One Touch Ultra, LifeScan Inc.). Area under the curve (AUC) for the GTT, ITT, and PTT was calculated by using Graphpad Prism Software™(La Jolla, CA).

### Construction and Purification of Adenoviruses Expressing KLF11 and KLF11 shRNA

Adenovirus expressing KLF11 (Ad-KLF11) or GFP (Ad-GFP), and short-hairpin RNA specific for KLF11 (Ad-shKLF11) or Short-hairpin RNA against luciferase (Ad-shCon) was generated as previously described [16].

### Transient Transfection and Luciferase Assays

HepG2 cells were grown in 24-well plates using Dulbecco’s Modified Eagle Medium (GIBCO, Grand Island, NY, USA) supplemented with 10% FBS in a humidified incubator in the presence of 5% CO_2_ at 37°C. Cells were co-transfected with human PEPCK-C promoter constructs (pGL3-PEPCK-C) and KLF11 expression plasmid or empty vectors (pcDNA3.1) using Lipofectamine™ 2000 (Invitrogen), according to the manufacture’s instructions. Renilar luciferase expression vector was co-transfected as an internal control. Luciferase activity was measured 48 hrs later with the Dual Luciferase Reporter Assay System™ (Promega, Madison, WI), followed with the manufacture’s instructions.

### Quantitative Real-time PCR (qRT-PCR)

Total RNA was isolated from cells or pulverized liver using TRIzol (Invitrogen). Primers were listed in Table S1.

### Western Blot Analysis

Protein was extracted from frozen organ samples or cultured hepatocytes in cell lysis buffer, and 40–60 µg of protein were loaded onto a 10% SDS PAGE and separated proteins were transferred to PVDF membranes. Membranes were probed with primary antibodies against PEPCK-C, Akt, p-Akt (Ser473), glycogen synthase kinase 3β (GSK3β), p-GSK3β (Cell Signaling Technology), c-Myc (Santa Cruz Biotechnology, Inc) and GAPDH (Abcam, Cambridge, MA). Protein on membrane was visualized by enhanced chemiluminescence reagent (GE Healthcare, Livonia, MI) and autoradiography.

### Glucose Output Assay

For glucose output assays, primary mouse hepatocytes were seeded in 6-well plates in RPMI-1640 containing 10% FBS, 100 units/ml penicillin, and 0.1 mg/ml streptomycin at 37°C in 15% atmospheric air/5% CO_2_. The next day, cells were infected with adenoviruses expressing GFP or KLF at an MOI of 100. Twenty-four hours after transduction, cells were washed 3 times with PBS. Cells were then incubated in 2 ml/well of phenol red–free, glucose-free DMEM containing 1 µM dexamethasone (Dex), 2 mM pyruvate, 20 mM lactate, and 10 µM forskolin (FSK). The medium was collected 3 hours later; an aliquot of 0.5 ml of medium was taken to measure the glucose concentration in the culture medium using a glucose assay kit (Applygen Technologies Inc.). A 2-fold concentration of the kit reagents was used to increase the sensitivity. Cells were collected and lysed, and the total protein concentration was measured (Bio-Rad, Hercules, Ca) to correct for the cell counts.

### In vivo Insulin Signaling

After an overnight fasting, mice were anesthetized with 2, 2, 2-tribromoethanol in PBS (Avertin) and injected with 5 U of regular human insulin (Sigma-Aldrich) via the inferior vena cava or IP injection. Five or ten minutes after the insulin bolus, livers were removed and frozen in liquid nitrogen. Immunoblot analysis of insulin signaling molecules was performed using tissue homogenates prepared in a tissue homogenization buffer (25 mM Tris-HCl (pH 7.4), 10 mM Na_3_VO_4_, 100 mM NaF, 50 mM Na_4_P_2_O_7_, 10 mM EGTA, 10 mM EDTA, 2 mM phenylmethylsulfonyl fluoride and 1% Nonidet-P40) supplemented with the Complete protease inhibitor cocktail (Roche).

### Statistical Analysis

Statistical analysis was performed using Graphpad Prism Software. Two-way ANOVA was used to compare the means between control and experimental group among different time points. Multiple row *t* test was performed to compare statistical difference between control and experimental group across different time points. Data are presented as means±SEM. * indicated the significant difference between compared groups (*P<0.05, **P<0.01, or ***P<0.001).

## Results

### Regulation of KLF11 Gene Expression in the Mouse Liver

**Figure 1:**
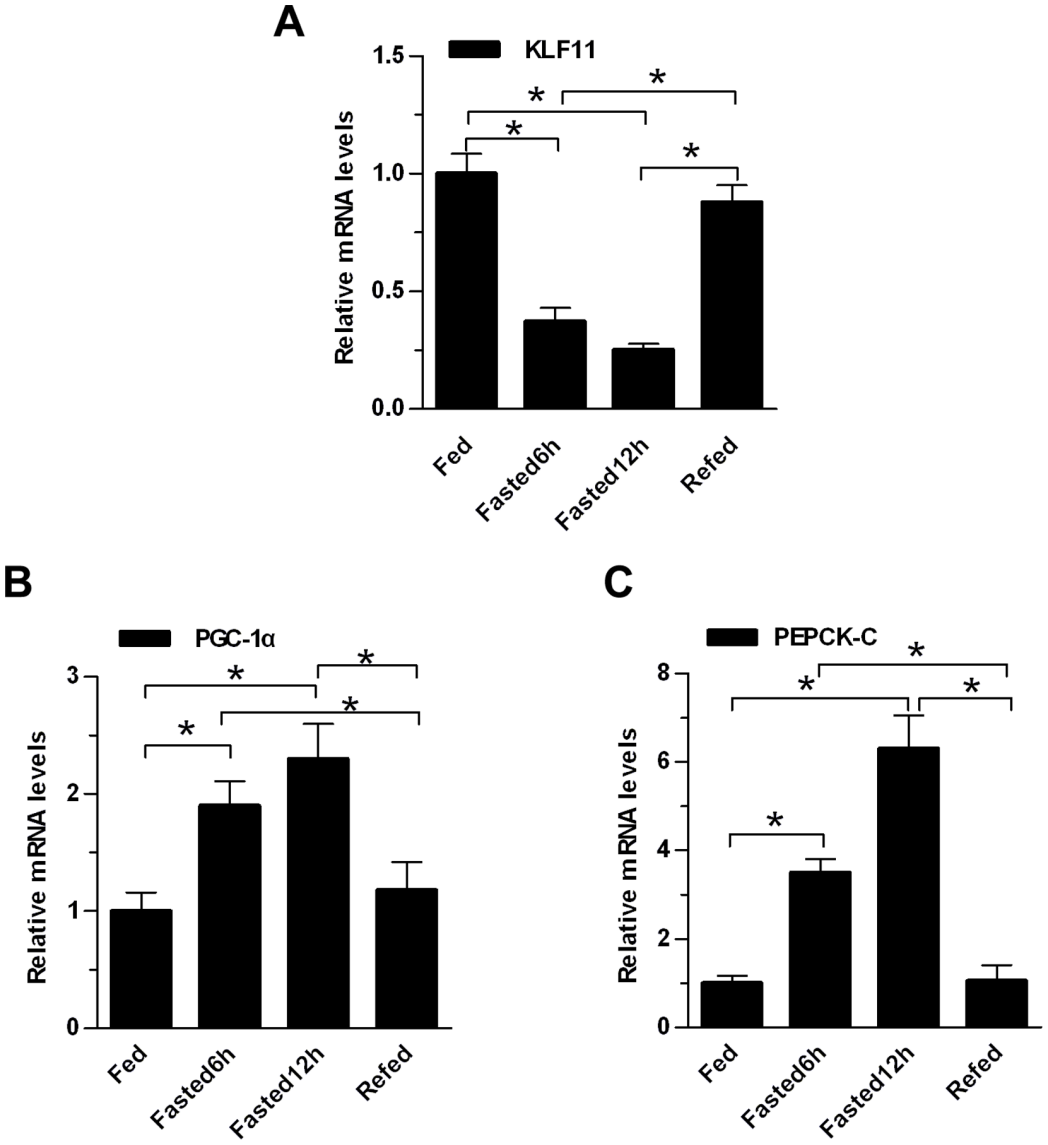
Regulation of KLF11 Gene Expression in the Mouse Liver. Quantitative real-time PCR analysis of mRNA levels of hepatic KLF11(A), PGC-1α (B), and PEPCK-C (C) in eight-week-old male C57BL/6J mice under *ad libitum* feeding, 6 hr fasting, 12 hr fasting and 12 hr fasted/12 hr refed conditions (n = 4/group). The data shown were the means ± SEM. Statistical significance was determined using a two-tailed Student’s t-test (*P<0.05, **P<0.01).

In previous work, we showed that hepatic KLF11 expression levels were decreased in db/db or DIO mice compared to control mice [16]. To determine whether KLF11 expression was regulated in the mouse livers in response to different nutritional status, male mice (7–8 weeks) were either short-term fasted (6-or 12-hr) or refed for 12-hr after fasting. Total hepatic RNA was prepared and KLF11 and key gluconeogenic genes expression was measured by qRT-PCR. Results suggested that short-term fasting decreased KLF11 expression levels, and refeeding restored its expression (Fig. 1A). In sharp contrast, PGC-1α and PEPCK-C exhibited the opposite expression pattern as KLF11 in the liver under identical nutritional conditions (Fig. 1B and C). These results suggested that hepatic KLF11 expression was regulated by metabolic signals. However, prolonged fasting (24 or 48 hours) induced KLF11 gene expression and this induction was reversed by refeeding. Furthermore, induced KLF11 stimulates fatty acid oxidation [16]. Of note, hepatic gluconeogenesis is very active during early fasting (6 hours), whereas it is markedly reduced and lipids oxidation is enhanced to provide ketone bodies during late fasting (18 hours) [17]. Based on these data, we speculated that KLF11 may negatively regulate hepatic gluconeogenesis. If that, expression pattern of KLF11 in the different fasting stage is actually consistent with the above physiological phenomenon.

### KLF11 Overexpression Inhibits PEPCK-C Promoter Activities in HepG2 Cells

**Figure 2:**
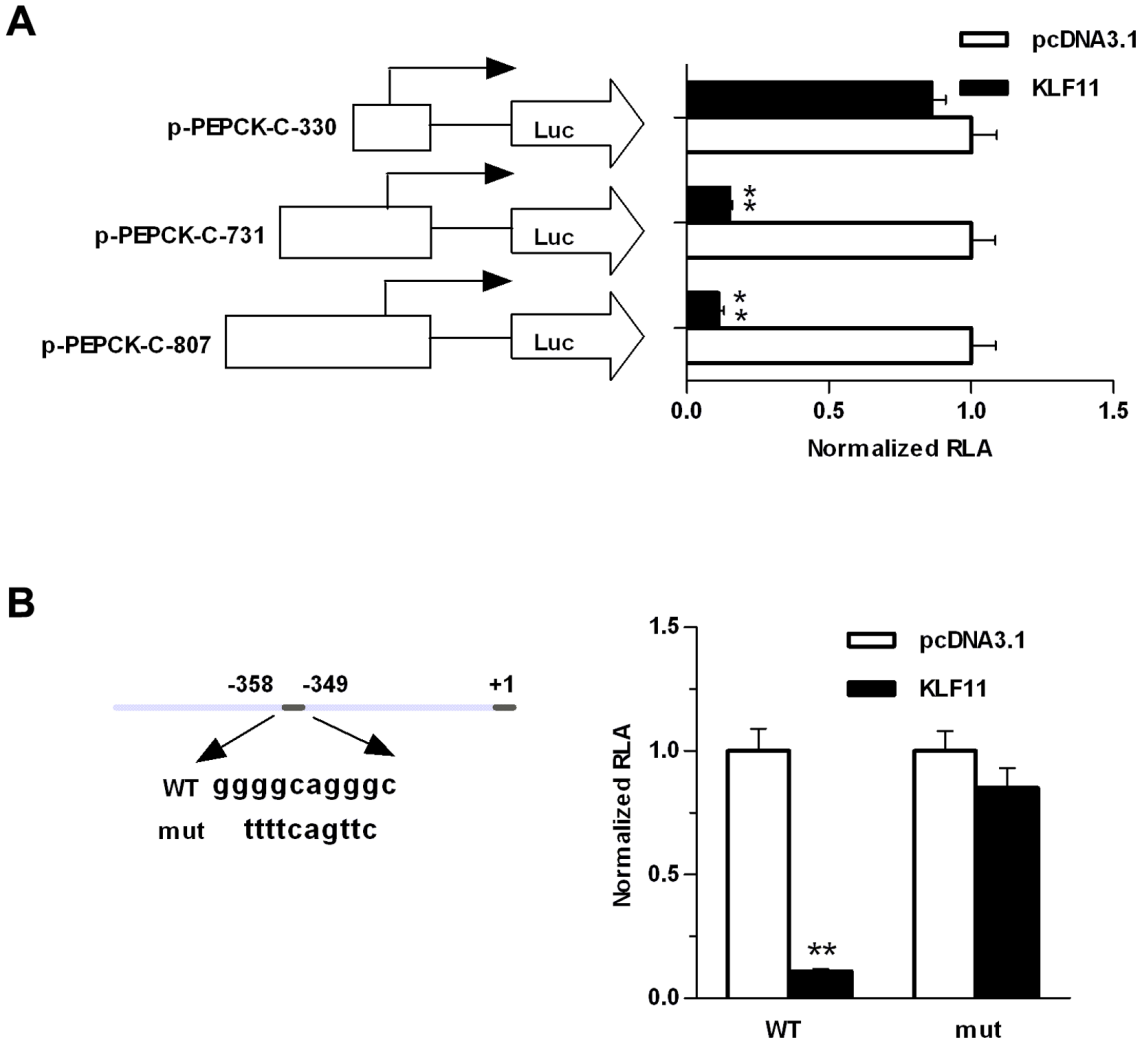
Functional analysis of the promoter region of PEPCK-C in HepG2 cells. (A) The longer human PEPCK-C promoter construct (pGL3-PEPCK-C-807) and 5′-deleted promoter constructs (pGL3-PEPCK-C-731, and pGL3-PEPCK-C-330) were co-transfected with pcDNA3.1-KLF11 expression plasmid into HepG2 cells, or with pcDNA3.1 (control). After 48 hours, relative luciferase activity (RLA) was measured. (B) pGL3-PEPCK-C-807 promoter construct and mutant construct (pGL3-PEPCK-C-mut) were transfected into HepG2 cells, together with pcDNA3.1-KLF11 expression plasmids or pcDNA3.1 (control). After 48 hours, relative luciferase activity (RLA) was measured. The data shown were the means ± SEM (n = 3). Statistical significance was determined using a two-tailed Student’s t-test (*P<0.05, **P<0.01).

We sought to explore the role of KLF11 in regulating the promoter activity of PEPCK-C. A bioinformatic prediction of transcription binding sites in PEPCK-C promoter was performed. In a ∼800 bp full-length PEPCK-C promoter sequence, we found multiple GC-rich sequences, which were described before maybe as KLF11 binding-site. Several shorter promoter fragments were generated, jumping of multiple GC-rich sequences in promoter. These promoter constructs were co-transfected with KLF11 expression vector to HepG2 cells. The luciferase activity suggested that overexpression of KLF11 inhibited the PEPCK-C promoter activity in HepG2 cells for both full-length and 731 bp promoter construct (Fig. 2A). However, the KLF11-mediated inhibition of PEPCK-C was abolished in a shorter fragment (∼330 bp, Fig. 2A), suggesting that the deleted promoter region was essential for KFL11-mediated inhibition. To delve into the mechanistic aspect of KLF11-mediated inhibition, a putative KLF11 binding-site was mutated in the full length promoter (Fig. 2B). The mutated promoter construct was co-trasnfected with KLF11 expression vector and KLF11 lost protestation to induce PEPCK-C promoter activity, suggesting that the mutated GC-rich sequence was important for KLF11-mediated induction (Fig. 2B).

### Overexpression of KLF11 Inhibits Gluconeogenic Program in HepG2 and Primary Hepatocytes

**Figure 3:**
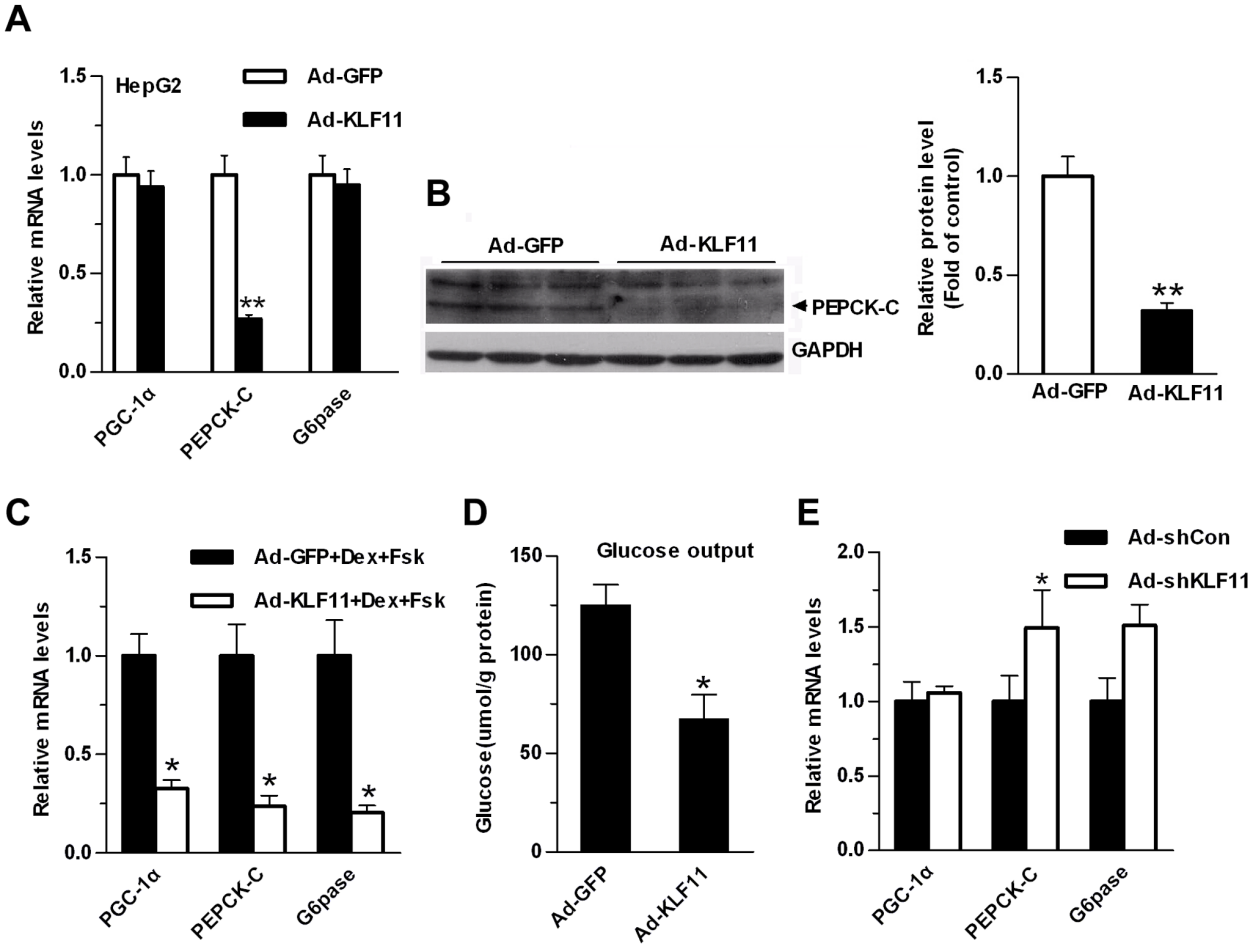
KLF11 regulates the expression of the gluconeogenic genes in HepG2 cells and mouse primary hepatocytes. (A) qRT-PCR analysis of the mRNA expression levels of gluconeogenic genes in HepG2 cells infected with adenoviruses Ad-GFP or Ad-KLF11. (B) Western blot analysis of the protein levels of PEPCK-C in HepG2 cells infected with Ad-GFP or Ad-KLF11 adenoviruses. GADPH was used to show the similar amount of protein loaded in different lanes (Left panel). The relative intensities of PEPCK-C bands on the Western blot were determined using NIH Image 1.62 software and normalized using GADPH band intensity (Right panel). (C) qRT-PCR analysis of the mRNA expression levels of gluconeogenic genes in primary hepatocytes infected with adenoviruses Ad-GFP or Ad-KLF11. At 24 hr after infection, hepatocytes were switched to starvation media for 6 hr, followed by treatment with 10 µM forskolin and 1 µM dexamethasone for 1.5 hr. (D) Measurement of cellular glucose production in primary hepatocytes as described in Fig. 3C. (E) qRT-PCR analysis showing the expression levels of gluconeogenic genes in primary hepatocytes infected with control Ad-shCon or Ad-shKLF11. Hepatocytes were grown for 2 days post-infection in RPMI-1640+10% FBS. The data shown were the means ± SEM (n = 3). Statistical significance was determined using a two-tailed Student’s t-test. * on each bar indicated the comparison between Ad-KFL11 and Ad-GFP infection (*P<0.05, **P<0.01).

KLF11 inhibited the PEPCK-C promoter activity and mutation of the GC-rich sequence in promoter sequence abolished KLF11-mediated suppression. Subsequently, we sought to pursue the functional importance of KLF11 in gluconeogenic program *in vitro*. HepG2 cells were infected with adenovirus expressing KLF11 or GFP (control). Overexpression of KLF11 decreased PEPCK-C expression level for both mRNA and protein (∼70%) in HepG2 cells (Fig. 3A), whereas the expression levels of PGC-1α and G6pase were not markedly affected (Fig. 3A and B). A similar experiment was performed in primary hepatocytes, although overexpression of KLF11 by Ad-KLF11 did not affect the expression of gluconeogenic genes in the primary hepatocytes under basal conditions (data not shown), it significantly inhibited the expression of these genes (including PGC-1α, PEPCK-C, and G6pase) in the presence of forskolin (Fsk) and dexamethasone (Dex), which mimic the fasting action of glucagons and glucocorticoids, respectively (Fig. 3C). The ability of KLF11 to inhibit the gluconeogenic program in hepatocytes suggested that it may decrease cellular glucose output. As expected, Ad-KLF11 infection reduced glucose production in primary mouse hepatocytes in the presence of FSK and Dex (Fig. 3D). In contrast, Ad-shKLF11-mediated reduction of KLF11 expression in primary hepatocytes modestly increased the expression levels of PEPCK-C and G6pase (Fig. 3E).

### KLF11 Ameliorates Hyperglycemia and Glucose Intolerance in Diabetic Mice

**Figure 4:**
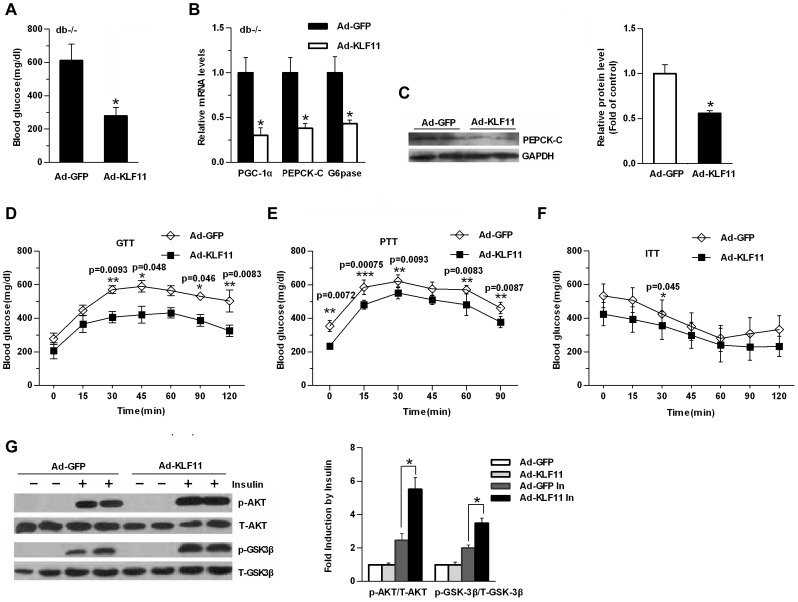
Overexpression of KLF11 in db/db mice alleviates hyperglycemia. (A) Blood glucose level in control Ad-GFP or Ad-KLF11-injected db/db mice 7 days after injection under fasting conditions (n = 6/group, 6-hr fasting). (B) qRT-PCR showing the mRNA levels of gluconeogenic genes in the livers of the same mice as in (A) (n = 6/group). (C) Western blot analysis showing the protein levels of PEPCK-C in in the livers of the same mice as in (A) (n = 6/group). GADPH was used to show the similar amount of protein loaded in different lanes (Left panel). The relative intensities of PEPCK-C bands on the Western blot were determined using NIH Image 1.62 software and normalized using GADPH band intensity (Right panel). (D–F) Glucose tolerance tests (GTTs) (D), pyruvate-tolerance tests (PTT) (E), Insulin tolerance tests (ITTs) (F) in control Ad-GFP or Ad-KLF11 -injected db/db mice 5 days after injection (n = 6/group). All data are presented as mean ± SEM, with statistical analysis performed by repeated-measures two-way ANOVA (*p<0.05, **P<0.01, ***P<0.001). (G) Db/db mice were injected with Ad-KLF11 or Ad-GFP. After 5 days, the mice were fasted overnight and anesthetized with tribromoethanol followed by IP injection of 5 U of insulin or saline (as a control). Ten minutes later, the animals were sacrificed, and their liver protein lysates were subjected to Western blot analysis (Left panel). The relative intensities of Phospho-Akt, total Akt, phosphor-GSK3β and total GSK3β were quantitated by densitometry analysis of their bands on film. The results are expressed as the ratios of phospho-Akt/total Akt and phosphor-GSK3β/total GSK3β (statistical analysis of Western blot data from 4 mice under each condition; Right panel). The data shown are the means ± SEM. Statistical significance was determined using a two-tailed Student’s t-test (*P<0.05, **P<0.01).

To determine whether KLF11 regulated hepatic gluconeogenesis *in vivo*, we performed genetic constitution experiments in db/db mice in which the endogenous expression of KLF11 was low. Our previous results indicated that exogenous myc-tagged KLF11 was effectively expressed in the liver mediated by adenovirus Ad-KLF11, but not in the other tissues, including muscle and adipose tissue [16]. We found that Ad-KLF11 injection of db/db mice had significantly lower fasting plasma glucose levels compared with control adenovirus (Ad-GFP) (Fig. 4A). Consistent with the decreased blood glucose levels, the expression of gluconeogenic genes, including PGC-1α and its downstream target genes PEPCK-C and G6pase, were decreased in the livers of Ad-KLF11-injected mice (Fig. 4B), and western blot analysis indicated that PEPCK-C protein levels were also decreased with KLF11 overexpression (Fig. 4C), indicating that the decrease in hepatic gluconeogenesis contributed to the lower glucose levels. Glucose tolerance tests (GTTs) showed that a modest induction of KLF11 in db/db mouse livers markedly improved glucose intolerance after an intraperitoneal glucose injection compared with control Ad-GFP-injected db/db mice (Fig. 4D). A pyruvate-tolerance test (PTT) demonstrated that *de novo* hepatic glucose production was reduced in Ad-KLF11-treated db/db mice (Fig. 4E). However, Insulin tolerance tests (ITTs) showed modestly increased insulin sensitivity in Ad-KLF11-infected db/db mice (Fig. 4F). AUC was reduced for GTT and PTT and ITT modestly decreased but did not reach statistical difference for ITT in mice with hepatic overexpression of KLF11 (Figure S1 A–C). These effects through KLF11 overexpression were accompanied by increased phosphorylation of Akt and its downstream target GSK3β in response to acute intraperitoneal insulin injections, whereas the total Akt and GSK3β protein levels remained unchanged (Figure 4G). These results suggest that the gain of function of KLF11 in the db/db mouse livers reduced hepatic glucose production, and improved glucose intolerance, but lesser extent insulin sensitivity.

### Hepatic Silencing of KLF11 Impairs Glucose Homeostasis in db/m and Wild-type C57BL/6J Mice

**Figure 5:**
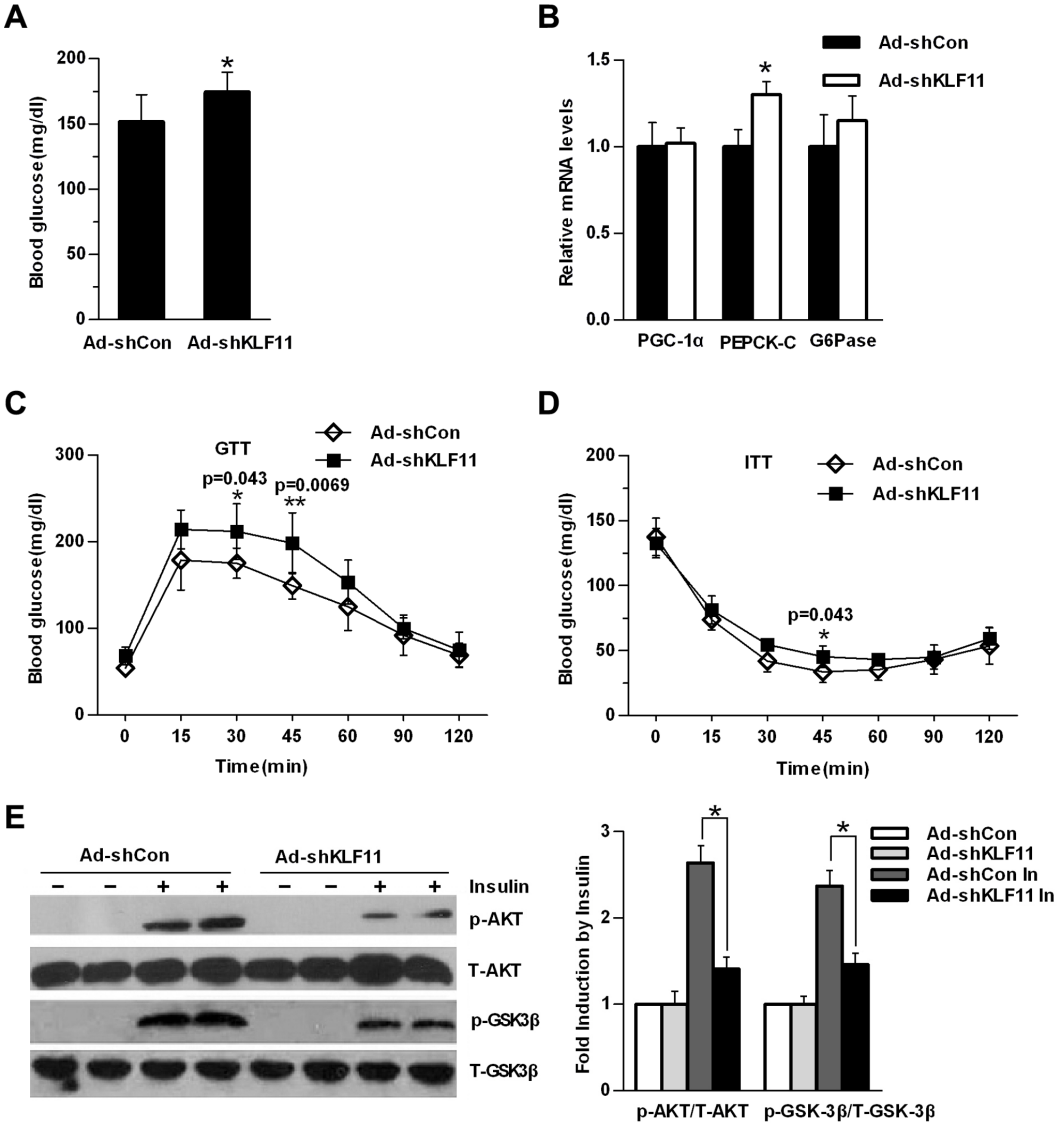
Knockdown of KLF11 in db/m mice livers impairs glucose homeostasis. (A) Blood glucose levels in control Ad-shCon- or Ad-shKLF11-injected db/m mice 7 days after injection under fasting conditions (n = 7/group, 6-hr fasting). (B) qRT-PCR analysis showing mRNA levels of gluconeogenic genes in the livers of the same mice as in (A) (n = 7/group). (C–D) Glucose tolerance tests (GTTs) (C) and insulin tolerance tests (ITTs) (D) in control Ad-shCon- or Ad-shKLF11-injected db/m mice 7 days after injection (n = 7/group are sure this is 7 not 6). All data are presented as mean ± SEM, with statistical analysis performed by repeated-measures two-way ANOVA (*p<0.05, **P<0.01). (E) Db/m mice were treated as described in (A). 5 days after adenovirus infection (ad-shKFL11 or ad-shCon), mice were fasted overnight and anesthetized with tribromoethanol followed with the injection of 5 U of insulin or saline (as a control) via the inferior vena cava. Five minutes later, the animals were sacrificed, and the liver protein lysates were subjected to western blot analysis. The relative intensities of insulin signaling molecules were quantitated by densitometry analysis of their bands on film. The results are expressed as the ratios of phospho-Akt/total Akt and phosphor-GSK3β/total GSK3β (statistical analysis of western blot data from 4 mice under each condition; Right panel). The data shown are the means ± SEM. Statistical significance was determined using Student’s t-test (*p<0.05, **p<0.01).

To further address the physiological roles of hepatic KLF11 expression in regulating glucose homeostasis, we injected an adenovirus expressing KLF11-specific shRNA (Ad-shKLF11) into db/m mice via a tail vein injection, which previously demonstrated an effective knockdown of hepatic KLF11 expression in mice [16]. Plasma fasting glucose levels in Ad-shKLF11-treated db/m mice tended to be higher than those in Ad-shCon-treated control mice (Fig. 5A). Consistent with these effects, knockdown of KLF11 modestly increased the hepatic expression levels of PEPCK-C and G6pase (Fig. 5B), indicating that the modestly increased blood glucose levels resulted in enhanced hepatic glucose production. GTTs and ITTs experiments showed that knockdown of KLF11 in db/m mouse livers significantly impaired glucose tolerance and barely affected insulin sensitivity, respectively (Fig. 5 C and D). AUC significantly increased in GTT and increased slightly but did not reach statistical difference for ITT in mice with hepatic knockdown of KLF11 (Figure S1 D and E). Meanwhile, these KLF11 knockdown-induced effects were accompanied by decreased phosphorylation of Akt and GSK3β in the liver in response to insulin (Fig. 5E).

We also performed KLF11 knockdown experiments in wild-type C57BL/6J mice. Although hepatic KLF11 knockdown in C57BL/6J mice did not markedly affect blood glucose levels (Figure S2A), it still significantly impaired glucose tolerance, as revealed by GTTs experiments (Figure S2C). However, ITTs experiments indicated that insulin sensitivity was barely affected in Ad-shKLF11-infected C57 mice (Figure S2D). Western blot analysis also confirmed that KLF11 knockdown inhibited insulin-stimulated phosphorylation of Akt and GSK3β (Figure S2E). These data indicated that KLF11 exerted a critical regulatory role in the glucose metabolism *in vivo*.

## Discussion

Despite the strong evidences linking KLF11 to Type 2 diabetes development [13]–[15], [18], the physiological functions of KLF11 *in vivo* remain largely unknown. In current study, we delved into the mechanistic aspect of KLF11 in hepatic glucose metabolism revealing that KFL11 played an essential role in regulating glucose homeostasis in liver. This conclusion was based on following results: (a) KLF11 and gluconeogenic genes expression level was regulated by fast-fed cycle in liver. (b) Modulation of the KFL11 expression in liver regulated gluconeogenic genes and affected glucose homeostasis. (c) KFL11 over-expression inhibited PEPCK-C promoter activity. (d) For truncated promoter construct (pGL3-PEPCK-C-330) with deletion of GC-rich sequence or longest promoter construct (pGL3-PEPCK-C-807) with mutation of GC-rich sequence, KFL11 over-expression lost inhibition effect.

Recent studies have shown that mutations in human KLF11 gene or KLF11 binding element in the human insulin promoter, which impairs KLF11 binding to promoter and activation of insulin gene promoter, results in diabetes leading to decreased human insulin biosynthesis [15], [19]. Moreover, fasting induces the expression of KLF11 in mouse skeletal muscles [20], and its promoter can be bound by hepatocyte nuclear factor-1α (HNF1-α) in hepatocytes [21]. KLF11, as a transcription factor, also directly binds to and activates uncoupling protein 1 (UCP-1) gene expression in brown adipocytes [22]. Our previous studies have shown that the expression levels of KLF11 decreased in db/db or DIO mouse livers compared with control mice [16]. Thus, these data implied that KLF11 might be involved in hepatic glucose homeostasis.

Initially, we speculated that the decreased KLF11 expression in diabetic mouse livers might contribute to diabetic phenotype. Thus, the restoration of KLF11 expression in diabetic mouse livers should improve glucose tolerance. We first characterized the KLF11 function in different cells. *In vitro* studies suggested that overexpression of KLF11 resulting in down-regulation of the expression of gluconeogenic genes such as PEPCK-C in HepG2 cells; however, the expression of the PGC-1α and G6pase was not significantly affected. In addition, it was observed that KLF11 overexpression significantly inhibited the expression of gluconeogenic genes in mouse primary hepatocytes, including PGC-1α, PEPCK-C and G6Pase, in the presence of Fsk and Dex, followed with decreased cellular glucose production.

KLF11 can function as either activator or repressor, depending on the cellular context in which the promoters it binds to and cofactor it recruits [11]. KLF11 and multiple KLF family members, including KLF10, KLF9 and KLF13, share a conserved repression motif, a α-helical domain highly related to the Sin3 interaction domain (SID) of the transcriptional repressor Mad1, at amino-terminal region. SID might mediate KLF11 repression activity by interacting with the histone deacetylase corepressor complex Sin3A [11], [13], [23]. Overexpression of KLF11 inhibits cell growth and suppresses neoplastic transformation and SID is required for these KLF11 effects [24]. However, recent studies suggested that KLF11 can also recruit coactivator p300 via its zinc finger domain to bind to and activate pancreatic-duodenal homeobox-1 (Pdx-1) gene promoter [18]. Likewise, KLF11 is a consistent activator of insulin promoter via a p300-mediated mechanism [15]. Our luciferase reporter assay demonstrated that KLF11 overexpression in HepG2 cells inhibited h-PEPCK-C gene promoter activity (for both pGL3-PEPCK-C-807 and pGL3-PEPCK-C-731, not pGL3-PEPCK-C-330). A GC-rich sequence at −358 to −349 was identified and mutated in the pGL3-PEPCK-C-807 promoter construct suggested the GC-rich sequence was essential for KLF11-mediated inhibition of PEPCK-C promoter activity. Therefore, chromatin immunoprecipitation (ChIP) assay was performed in HepG2 cells with KLF11 overexpression. However, the result did not indicate a direct interaction between KLF11 and PEPCK-C promoter (data not shown). We also tested the inhibition effect of KLF11 on mouse PEPCK-C and PGC-1α promoter activity showing similar trend of inhibition (data not shown), although to a lesser extent, which indicated an evolutionary conserved function of KFL11 in hepatic gluconeogenesis.

Meanwhile, forced expression of KLF11 by adenovirus in db/db diabetic mice livers significantly decreases blood glucose levels even in a mild trend. However, overexpression of KLF11 did not significantly altered plasma insulin levels. It is notable that activation of KLF11 in the DIO mice liver did not significantly affect blood glucose levels (Figure S3), reflecting the differences in genetic backgrounds between these two types of mice. Conversely, loss of KLF11 function in db/m mice modestly increased blood glucose levels and impaired glucose tolerance, but not insulin tolerance. In addition, knockdown of KLF11 did not significantly affect the expression of gluconeogenic genes. We also performed KLF11 knockdown experiments in C57BL/6J mice and obtained similar results. Interestingly, signal transducer activator of transcription 3 (STAT3) is identified to directly bind to and activate wild-type KLF11 gene promoter, whereas STAT3 did not bind to KLF11 mutant promoter [25]. Mice with liver-specific knockout of STAT3 display insulin resistance associated with increased expression of hepatic gluconeogenic genes, such as PEPCK-C and G6Pase. It is possible that KLF11, as a downstream target gene of STAT3, mediates the effects of STAT3 on the expression of gluconeogenic genes [26].

In conclusion, our study revealed the physiological role of KLF11 in regulating hepatic glucose metabolism. Activation of KLF11 in mouse livers decreased expression levels of PEPCK-C both in mRNA and protein, thereby inhibiting hepatic glucose production and decreasing blood glucose levels.

## Supporting Information

Figure S1 **The area under the curbe in GTT, ITT and PTT studies.** (A–C) The AUC of GTT(A), PTT(B) and ITT(C) for control Ad-GFP or Ad-KLF11 -injected db/db mice 5 days after injection (n = 6/group). (D–E) The AUC of GTT(D) and ITT(E) for control Ad-shCon- or Ad-shKLF11-injected db/m mice 7 days after injection (n = 7/group). All data are presented as mean ± SEM, with statistical analysis performed by two-tailed Student’s t-test (*p<0.05, *p<0.001, ***p<0.001).
(DOC)

Figure S2 **Knockdown of KLF11 in C57BL/6J mice livers impairs glucose tolerance.** (A) Blood glucose levels in control Ad-shCon- or Ad-shKLF11-injected C57BL/6J mice 7 days after injection under fasting conditions (n = 7/group). (B) Quantitative real-time PCR analysis showing the mRNA levels of gluconeogenic genes in the livers of the same mice as in (A) (n = 7/group). (C) Glucose tolerance tests (GTTs) (left) and the AUC of GTT (right) in control Ad-shCon- or Ad-shKLF11-injected C57BL/6J mice 7 days after injection (n = 7/group). (D) Insulin tolerance tests (ITTs) (left) and the AUC of GTT (right) in control Ad-shCon- or Ad-shKLF11-injected C57BL/6J mice 7 days after injection (n = 7/group). All data are presented as mean ± SEM, with statistical analysis performed by repeated-measures two-way ANOVA and two-tailed Student’s t-test (*p<0.05, **p<0.01). (E) C57BL/6J mice were treated as described in (A). After 5 days, the mice were fasted overnight and anesthetized with tribromoethanol followed by the injection of 5 U of insulin or saline (as a control) via the inferior vena cava. Five minutes later, the animals were sacrificed, and the liver protein lysates were subjected to western blot analysis (Left panel). The relative intensities of insulin signaling molecules were quantitated by densitometry analysis of their bands on film. The results are expressed as the ratios of phospho-Akt/total Akt and phosphor-GSK3β/total GSK3β (statistical analysis of Western blot data from 4 mice under each condition; Right panel). The data shown are the means ± SEM. Statistical significance was determined using a two-tailed Student’s t-test (*p<0.05, **p<0.01).
(DOC)

Figure S3 **Blood glucose levels in control Ad-GFP- or Ad-KLF11-injected High-fat diet-induced (HFD) obese mice 7 days after injection under fasting conditions (n = 6/group).**
(DOC)

Table S1 **RT-PCR primers.** All primers are listed in 5′ to 3′ direction (mouse-m, human-h).
(DOC)
