# Supplementary material for: Involvement of KLF11 in Hepatic Glucose Metabolism in Mice via Suppressing of PEPCK-C Expression
Source: PLoS One. 2014 Feb 26;9(2):e89552. doi: 10.1371/journal.pone.0089552 (PMC3935883; doi:10.1371/journal.pone.0089552)
Supplement: Figure S1 — The area under the curbe in GTT, ITT and PTT studies. (A–C) The AUC of GTT(A), PTT(B) and ITT(C) for control Ad-GFP or Ad-KLF11 -injected db/db mice 5 days after injection (n = 6/group). (D–E) The AUC of GTT(D) and ITT(E) for control Ad-shCon- or Ad-shKLF11-injected db/m mice 7 days after injection (n = 7/group). All data are presented as mean ± SEM, with statistical analysis performed by two-tailed Student’s t-test (*p<0.05, *p<0.001, ***p<0.001). (DOC) [file pone.0089552.s001.doc]

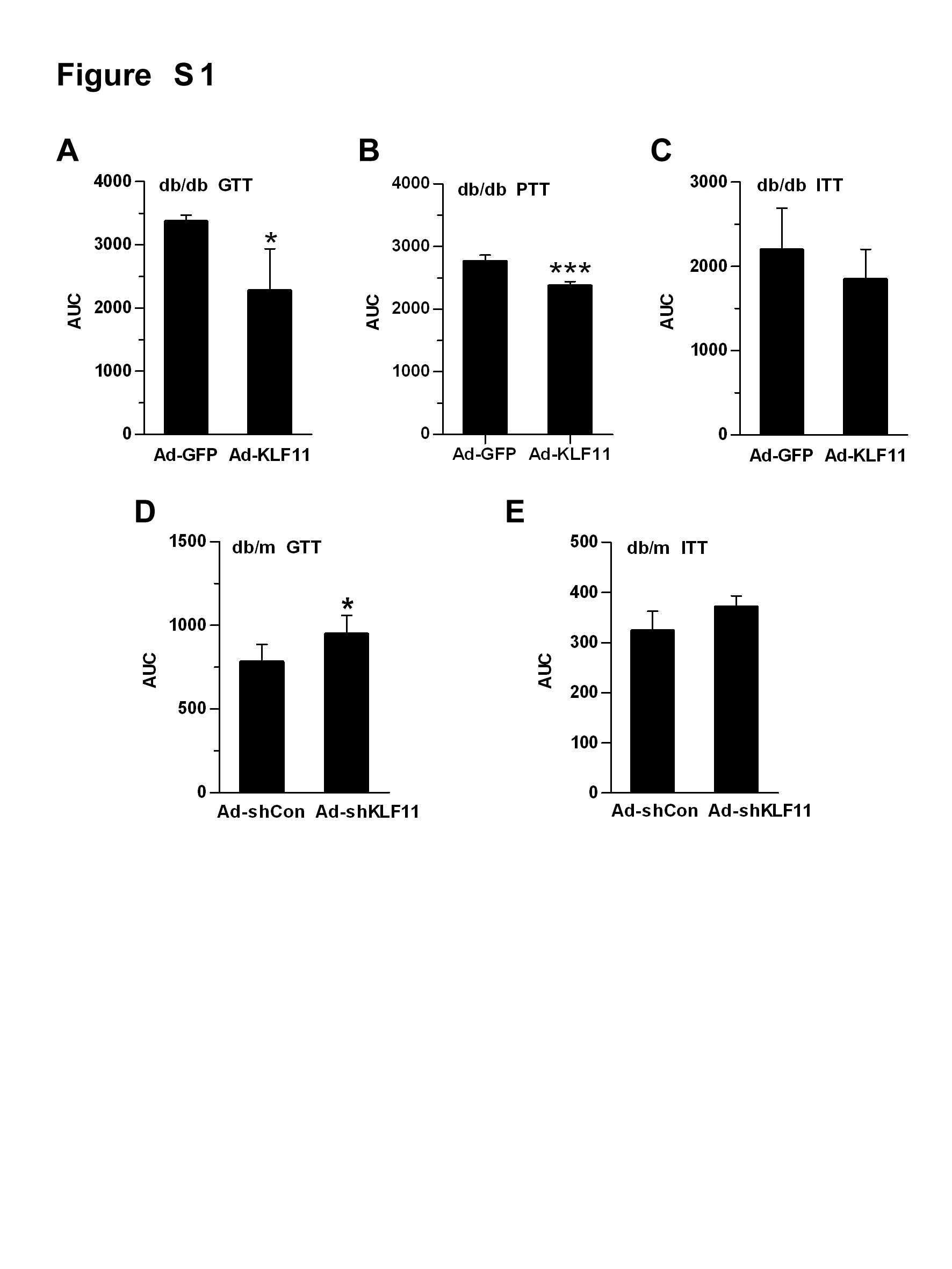


**Figure S1 The area under the curbe in GTT, ITT and PTT studies**

(A-C) The AUC of GTT(A), PTT(B) and ITT(C) for control Ad-GFP or Ad-KLF11 -injected db/db mice 5 days after injection (n=6/group).

(D-E) The AUC of GTT(D) and ITT(E) for control Ad-shCon- or Ad-shKLF11-injected db/m mice 7 days after injection (n=7/group).

All data are presented as mean ± SEM, with statistical analysis performed by two-tailed Student’s t-test (*p<0.05, *p<0.001, ***p<0.001).
