# Supplementary material for: Involvement of KLF11 in Hepatic Glucose Metabolism in Mice via Suppressing of PEPCK-C Expression
Source: PLoS One. 2014 Feb 26;9(2):e89552. doi: 10.1371/journal.pone.0089552 (PMC3935883; doi:10.1371/journal.pone.0089552)
Supplement: Figure S2 — Knockdown of KLF11 in C57BL/6J mice livers impairs glucose tolerance. (A) Blood glucose levels in control Ad-shCon- or Ad-shKLF11-injected C57BL/6J mice 7 days after injection under fasting conditions (n = 7/group). (B) Quantitative real-time PCR analysis showing the mRNA levels of gluconeogenic genes in the livers of the same mice as in (A) (n = 7/group). (C) Glucose tolerance tests (GTTs) (left) and the AUC of GTT (right) in control Ad-shCon- or Ad-shKLF11-injected C57BL/6J mice 7 days after injection (n = 7/group). (D) Insulin tolerance tests (ITTs) (left) and the AUC of GTT (right) in control Ad-shCon- or Ad-shKLF11-injected C57BL/6J mice 7 days after injection (n = 7/group). All data are presented as mean ± SEM, with statistical analysis performed by repeated-measures two-way ANOVA and two-tailed Student’s t-test (*p<0.05, **p<0.01). (E) C57BL/6J mice were treated as described in (A). After 5 days, the mice were fasted overnight and anesthetized with tribromoethanol followed by the injection of 5 U of insulin or saline (as a control) via the inferior vena cava. Five minutes later, the animals were sacrificed, and the liver protein lysates were subjected to western blot analysis (Left panel). The relative intensities of insulin signaling molecules were quantitated by densitometry analysis of their bands on film. The results are expressed as the ratios of phospho-Akt/total Akt and phosphor-GSK3β/total GSK3β (statistical analysis of Western blot data from 4 mice under each condition; Right panel). The data shown are the means ± SEM. Statistical significance was determined using a two-tailed Student’s t-test (*p<0.05, **p<0.01). (DOC) [file pone.0089552.s002.doc]

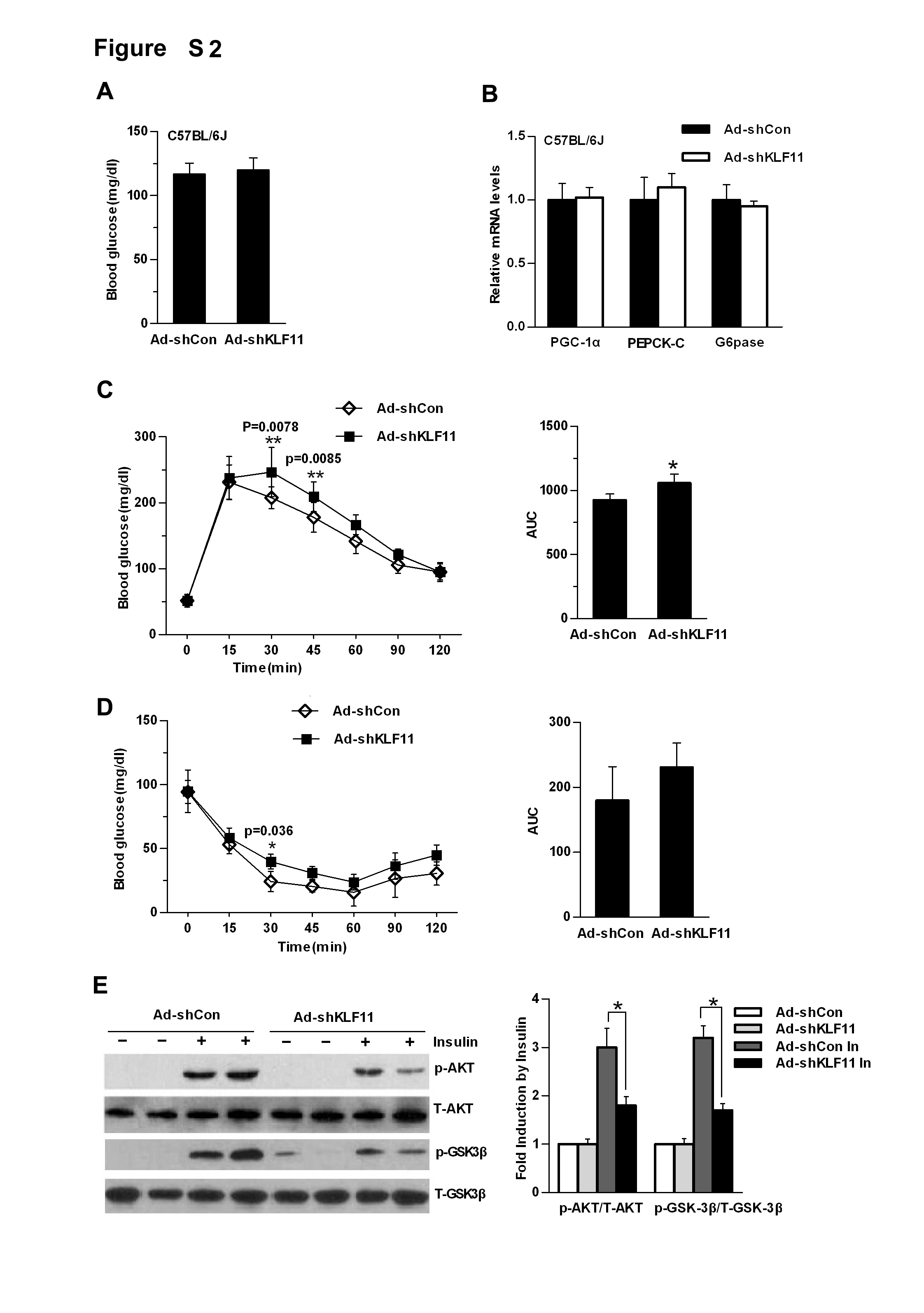


**Figure S2 Knockdown of KLF11 in C57BL/6J mice livers impairs glucose tolerance**

(A) Blood glucose levels in control Ad-shCon- or Ad-shKLF11-injected C57BL/6J mice 7 days after injection under fasting conditions (n=7/group).

(B) Quantitative real-time PCR analysis showing the mRNA levels of gluconeogenic genes in the livers of the same mice as in (A) (n=7/group).

(C) Glucose tolerance tests (GTTs) (left) and the AUC of GTT (right) in control Ad-shCon- or Ad-shKLF11-injected C57BL/6J mice 7 days after injection (n=7/group).

(D) Insulin tolerance tests (ITTs) (left) and the AUC of GTT (right) in control Ad-shCon- or Ad-shKLF11-injected C57BL/6J mice 7 days after injection (n=7/group). All data are presented as mean ± SEM, with statistical analysis performed by repeated-measures two-way ANOVA and two-tailed Student’s t-test (*p<0.05, **p<0.01).

(E) C57BL/6J mice were treated as described in (A). After 5 days, the mice were fasted overnight and anesthetized with tribromoethanol followed by the injection of 5 U of insulin or saline (as a control) via the inferior vena cava. Five minutes later, the animals were sacrificed, and the liver protein lysates were subjected to western blot analysis (Left panel). The relative intensities of insulin signaling molecules were quantitated by densitometry analysis of their bands on film. The results are expressed as the ratios of phospho-Akt/total Akt and phosphor-GSK3β/total GSK3β (statistical analysis of Western blot data from 4 mice under each condition; Right panel).

The data shown are the means ± SEM. Statistical significance was determined using a two-tailed Student’s t-test (*p < 0.05, **p < 0.01).
