# Supplementary material for: Involvement of KLF11 in Hepatic Glucose Metabolism in Mice via Suppressing of PEPCK-C Expression
Source: PLoS One. 2014 Feb 26;9(2):e89552. doi: 10.1371/journal.pone.0089552 (PMC3935883; doi:10.1371/journal.pone.0089552)
Supplement: Figure S3 — Blood glucose levels in control Ad-GFP- or Ad-KLF11-injected High-fat diet-induced (HFD) obese mice 7 days after injection under fasting conditions (n = 6/group). (DOC) [file pone.0089552.s003.doc]

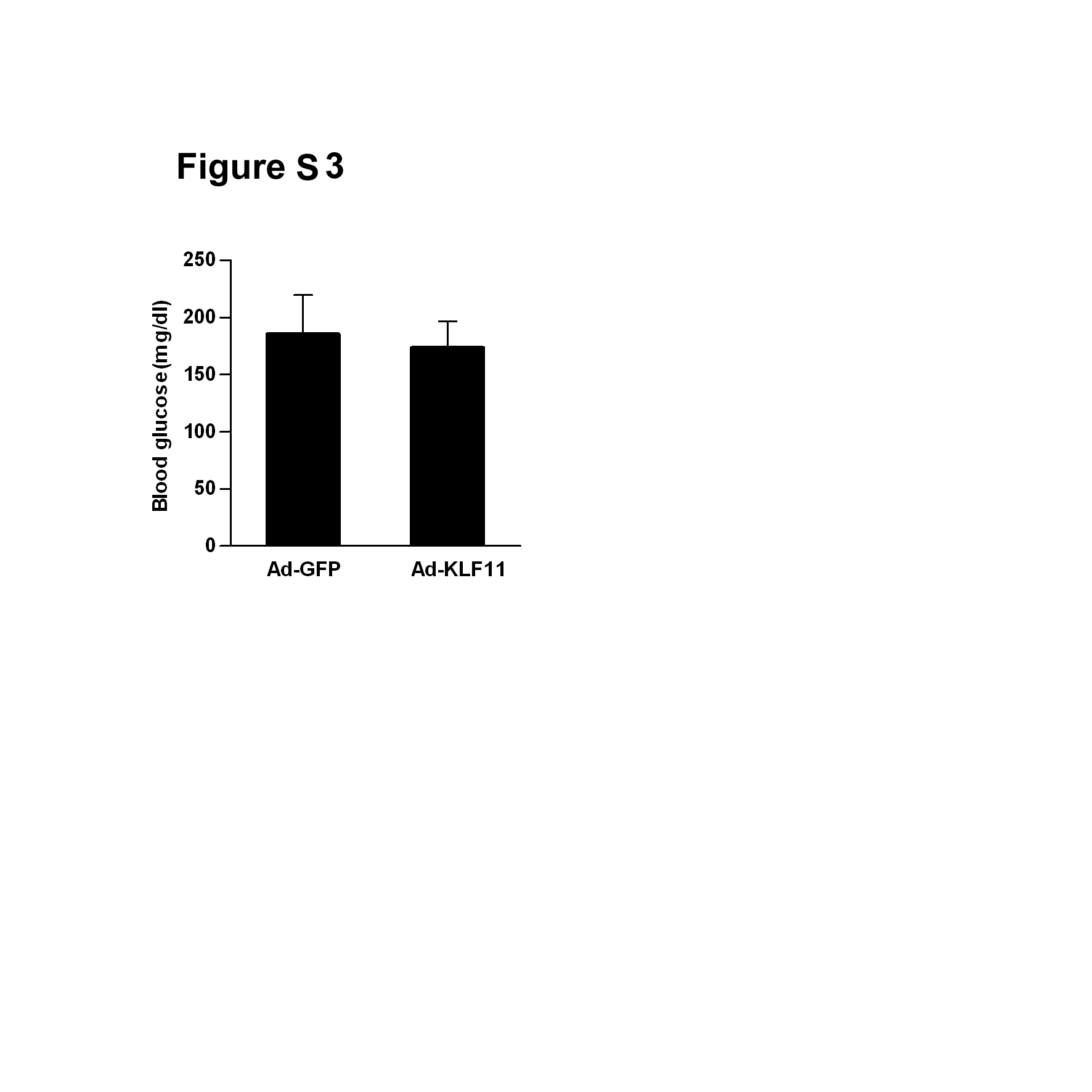


**Figure S3** Blood glucose levels in control Ad-GFP- or Ad-KLF11-injected High-fat diet-induced (HFD) obese mice 7 days after injection under fasting conditions (n=6/group).
