# Supplementary material for: Involvement of KLF11 in Hepatic Glucose Metabolism in Mice via Suppressing of PEPCK-C Expression
Source: PLoS One. 2014 Feb 26;9(2):e89552. doi: 10.1371/journal.pone.0089552 (PMC3935883; doi:10.1371/journal.pone.0089552)
Supplement: Table S1 — RT-PCR primers. All primers are listed in 5′ to 3′ direction (mouse-m, human-h). (DOC) [file pone.0089552.s004.doc]

**Table S1: RT-PCR primers**

All primers are listed in 5’ to 3’ direction (mouse-m, human-h).

| gene | Forward Primer | Reverse Primer |
| --- | --- | --- |
| m-KLF11 | GTGCCCCTCAGGTAGACTT | CACAAACTTCTTCTCTCCC |
| m-PGC-1α | TGGACGGAAGCAATTTTTCA | TTACCTGCGCAAGCTTCTCT |
| h-PGC-1α | GTCCTTCCTCCATGCCTGAC | CTGGGTACTGAGACCACTGC |
| m-PEPCK | CAGGATCGAAAGCAAGACAGT | ATCTTGCCCTTGTGTTCTGC |
| h-PEPCK | GGTTCCCAGGGTGCATGAAA | CACGTAGGGTGAATCCGTCAG |
| m-G6pase | AAGTCCTCTTCCGACATCCAG | GTCTCACAGGTGACAGGGAAC |
| h-G6pase | AGTTCCCTGTAACCTGTGAG | ATCTGTAGGTCGGCTTTATC |
| m-β-actin | AAATCGTGCGTGACATCAAA | AAGGAAGGCTGGAAAAGAGC |
| m-GAPDH | AAATGGTGAAGGTCGGTGTG | CATGTAGTTGAGGTCAATGAAGG |
| h-β-actin | AGCAGCATCCCCCAAAGTT | GGGCACGAAGGCTAATCATT |
